# Supplementary material for: Bacterial Pulmonary Co-Infections on ICU Admission: Comparison in Patients with SARS-CoV-2 and Influenza Acute Respiratory Failure: A Multicentre Cohort Study
Source: Biomedicines. 2022 Oct 20;10(10):2646. doi: 10.3390/biomedicines10102646 (PMC9599916; doi:10.3390/biomedicines10102646)
Supplement: Supplementary file 1 [file biomedicines-10-02646-s001.zip › biomedicines-1967134-supplementary.pdf]

Table S1: Results of studies comparing co-infections in patients with influenza or SARS-CoV2 pneumonia or comparing the two diseases.

	Authors, year	Type of study	% co-infections	Pathogens	Risk factors	Added mortality risk
**Influenza**	**(MacIntyre et al. 2018)**	Review	19–47%	*S. pneumoniae* (54% hospit, 24% ICU), *A. baumannii* (5-21%), MRSA (3-6%), *K. pneumoniae* (1-8%)		
	**(Mercat et al. 2011)**	Review	30% in ICU	*S. pneumoniae* (46%), *S. aureus* (20%)	None found	22% vs. 20%. Especially if co-inf with GNB and if cormorbity
	**(Rice et al. 2012)**	Retrospective, US, multicentric	154/683 (22.5%)	*S. aureus* (37%) *S. pneumoniae* (12%)		S aureus (relative risk 2.82; 95% confidence interval 1.76-4.51; p< 0.01)
**SARS-CoV2**	**(Westblade, Simon & Satlin 2021)**	Review	4%	*S. aureus* (31%), *S. pneumoniae* (23%), *H. influenzae* (11%)	Age, chronic kidney and heart diseases and diabetes	↗ duration of hospitalisation (7 days vs. 5 $p = 0.003$) and hospital mortality (48% vs. 18% $p < 0.001$)
	**(Baskaran et al. 2021)**	Observational, UK, multicentric	5.5%	*S. aureus* (31%), *S. pneumoniae* (23%)		
	**(Garcia-Vidal et al. 2021)**	Retrospective cohort, Barcelona	7.2% and 3.1% early co-infections	*S. pneumoniae* (12 cases), *S. aureus* (12), *Pseudomonas aeruginosa* (10), *Escherichia coli* (7), and *Klebsiella pneumoniae* (6)	Diabetes, chronic kidney and heart diseases	No added mortality for early co-infections
	**(Musuuza et al. 2021)**	Meta-analysis	18% in ICU	*K. pneumoniae* (9.9%), *S. pneumoniae* (8.2%), and *S. aureus* (7.7%).		Added risk of mortality if co-infection (OR = 2.84; 95% CI: 1.42–5.66)
	**(Rawson et al. 2020)**	Review	8%	No data	No data	No data
	**(Contou et al. 2020)**	Retrospective, France, monocentric	19/92(20%)	*S. aureus* (31.6%), *H. Influenzae* (31.6%), *S Pneumoniae* (26%), Enterobacter spp. (26%)	No data	No data
	**(De Santis et al. 2022)**	Retrospective, Italy, multicentric	35/245 (14.3%)	*S. aureus (48.5%), H Influenzae (20%)*	No data	No data

	**(Elabbadi et al. 2021)**	Retrospective, France, monocetric	20/101(19.8%)	*S. aureus (55%)* Enterobacter spp. *(40%)*	No data	No data
	**(Russel et al. 2021)**	Retrospective, UK, multicentric, Hospital admission	318/48 902	S. aureus (17.8%), H. influenzae (12.7%) and P. aeruginosa (9.3%) were most frequently identified in positive sputum samples, S. aureus in positive deep respiratory samples (31.1%), and E Coli (26.7%) and S aureus (13.3%) in positive blood cultures	No data	found no association in patients admitted to critical care who were identified to have a respiratory or bloodstream infection and subsequent mortality (unadjusted odds ratio 1.02, 95% CI 0.86–1.22; p = 0.81)
	Santos et al. 2022	Meta-analysis	1394 co-infected patients.	Enterobacter spp. (35%), S aureus (27%), Klebsiella spp. (21%)		
**Influenza and SARS-CoV2**	**(Rouze et al. 2021)**	Retrospective, France, multicentric	9.7% (SARS-CoV2) vs. 33.6% (Influenza)	GPCs responsable for 58% and 72% of co-infections and GNBs for 41.8% and 27.8% in patients with SARS-CoV2 and with influenza, respectively.	None found	No difference in impact on mortality
	**(Hedberg et al. 2022)**	Retrospective, monocentric	4% (SARS-CoV2) vs. 27% (Influenza)	No difference, *S. pneumoniae* and *S. aureus*	None found	No difference in mortality
	**(Pandey et al. 2022)**	Retrospective, multicentric retrospective cohort	8.7% (SARS-CoV2) vs. 25% (Influenza)	*S. aureus* foremost in 2 groups	None found	No data
	**(Sarton et al. 2022)**	Retrospective, France, monocentric	11/65 (16) (SARS-CoV2) vs 20/60 (33) (Influenza)	Influenza: *S aureus* (40%), *S pneumoniae* (22%); COVID-19: *S aureus* (25%), *H influenzae* (25%), non fermenting gram-negative bacilli (41%), *Enterobacteriaceae* (8%)	No data	No data

Table S2: Comparison of living and deceased patients in influenza and/or SARS-CoV2 pneumonia at D60.

	**All**					**Influenza**					**SARS-CoV2**				
	Living	Deceased	HR	95% CI	*p*	Living	Deceased	HR	95% CI	*p*	Living	Deceased	HR	95% CI	*p*
**Number of patients**	965	384	.			129	28	.			836	356	.		
**Time from admission to ICU > 2 days**	369 (38.2)	157 (40.9)	1.09	[0.89 ; 1.34]	0.40	28 (21.7)	10 (35.7)	1.59	[0.73 ; 3.46]	0.24	341 (40.8)	147 (41.3)	1.00	[0.81 ; 1.24]	1.00
**Age > 60 years**	506 (52.4)	317 (82.6)	3.21	[2.46 ; 4.18]	<0.01	62 (48.1)	17 (60.7)	1.64	[0.76 ; 3.54]	0.20	444 (53.1)	300 (84.3)	3.34	[2.5 ; 4.45]	<0.01
**Sex (male)**	690 (71.5)	267 (69.5)	0.86	[0.7 ; 1.08]	0.19	78 (60.5)	15 (53.6)	0.67	[0.32 ; 1.42]	0.30	612 (73.2)	252 (70.8)	0.83	[0.66 ; 1.05]	0.11
**BMI > 30 kg/m²**	368 (38.1)	140 (36.5)	0.93	[0.76 ; 1.15]	0.51	34 (26.4)	11 (39.3)	1.47	[0.69 ; 3.16]	0.32	334 (40)	129 (36.2)	0.86	[0.7 ; 1.07]	0.19
**Chronic cardiovascular disease**	193 (20)	135 (35.2)	1.89	[1.53 ; 2.34]	<0.01	18 (14)	4 (14.3)	1.40	[0.47 ; 4.16]	0.55	175 (20.9)	131 (36.8)	1.78	[1.43 ; 2.21]	<0.01
**Chronic lung disease**	121 (12.5)	61 (15.9)	1.08	[0.82 ; 1.42]	0.59	42 (32.6)	7 (25)	0.65	[0.28 ; 1.54]	0.33	79 (9.4)	54 (15.2)	1.36	[1.02 ; 1.83]	0.04
**Chronic kidney disease**	67 (6.9)	59 (15.4)	1.93	[1.45 ; 2.55]	<0.01	14 (10.9)	4 (14.3)	1.42	[0.49 ; 4.09]	0.52	53 (6.3)	55 (15.4)	2.00	[1.49 ; 2.68]	<0.01
**Immunosuppression**	132 (13.7)	73 (19)	1.21	[0.94 ; 1.57]	0.15	42 (32.6)	17 (60.7)	2.69	[1.25 ; 5.81]	0.01	90 (10.8)	56 (15.7)	1.26	[0.95 ; 1.68]	0.11
**Chronic liverc disease**	18 (1.9)	15 (3.9)	1.68	[1 ; 2.82]	0.05	5 (3.9)	2 (7.1)	1.29	[0.3 ; 5.45]	0.73	13 (1.6)	13 (3.7)	1.97	[1.13 ; 3.43]	0.02
**Diabetes**	142 (14.7)	68 (17.7)	1.18	[0.91 ; 1.53]	0.22	20 (15.5)	6 (21.4)	1.24	[0.5 ; 3.1]	0.64	122 (14.6)	62 (17.4)	1.17	[0.89 ; 1.55]	0.25
**SAPS > 40**	233 (24.1)	207 (53.9)	2.39	[1.95 ; 2.92]	<0.01	54 (41.9)	22 (78.6)	3.88	[1.56 ; 9.63]	<0.01	179 (21.4)	185 (52)	2.43	[1.97 ; 2.99]	<0.01
**SOFA > 8**	86 (8.9)	100 (26)	2.47	[1.96 ; 3.13]	<0.01	19 (14.7)	11 (39.3)	2.73	[1.26 ; 5.9]	0.01	67 (8)	89 (25)	2.56	[2 ; 3.28]	<0.01
**PaO_2/FiO_2 < 150**	617 (63.9)	291 (75.8)	1.46	[1.16 ; 1.85]	<0.01	71 (55)	17 (60.7)	1.26	[0.59 ; 2.71]	0.55	546 (65.3)	274 (77)	1.42	[1.11 ; 1.82]	0.01
**Invasive mechanical ventilation**	262 (27.2)	162 (42.3)	1.39	[1.13 ; 1.72]	<0.01	62 (48.1)	17 (60.7)	1.17	[0.54 ; 2.53]	0.69	200 (23.9)	145 (40.8)	1.64	[1.31 ; 2.04]	<0.01
**Vasopressors**	144 (14.9)	105 (27.3)	1.76	[1.38 ; 2.25]	<0.01	12 (9.3)	6 (21.4)	2.11	[0.84 ; 5.25]	0.11	132 (15.8)	99 (27.8)	1.64	[1.27 ; 2.12]	<0.01
**Renal replacement therapy**	22 (2.3)	32 (8.3)	2.64	[1.83 ; 3.8]	<0.01	7 (5.4)	2 (7.1)	1.41	[0.33 ; 5.99]	0.64	15 (1.8)	30 (8.4)	2.94	[2.02 ; 4.29]	<0.01
**Corticoids**	513 (53.2)	229 (59.6)	1.18	[0.95 ; 1.46]	0.13	35 (27.1)	4 (14.3)	0.39	[0.14 ; 1.14]	0.09	478 (57.2)	225 (63.2)	1.14	[0.91 ; 1.43]	0.26
**Antibiotics at admission**	539 (55.9)	246 (64.1)	1.21	[0.98 ; 1.51]	0.08	67 (51.9)	14 (50)	1.19	[0.56 ; 2.52]	0.65	472 (56.5)	232 (65.2)	1.19	[0.95 ; 1.49]	0.13
**Beta-lactamase inhibitor**	106 (11)	62 (16.1)	1.50	[1.14 ; 1.98]	<0.01	32 (24.8)	11 (39.3)	1.84	[0.86 ; 3.95]	0.12	74 (8.9)	51 (14.3)	1.82	[1.34 ; 2.48]	<0.01

**Cephalosporin**	412 (42.7)	182 (47.4)	1.02	[0.83 ; 1.26]	0.82	40 (31)	9 (32.1)	1.26	[0.57 ; 2.8]	0.57	372 (44.5)	173 (48.6)	0.96	[0.77 ; 1.18]	0.68
**Macrolides**	271 (28.1)	109 (28.5)	0.97	[0.77 ; 1.22]	0.81	34 (26.4)	9 (32.1)	1.75	[0.78 ; 3.94]	0.17	237 (28.3)	100 (28.2)	0.94	[0.74 ; 1.19]	0.59
**Aminosides**	30 (3.1)	37 (9.6)	2.30	[1.64 ; 3.24]	<0.01	7 (5.4)	6 (21.4)	4.42	[1.74 ; 11.22]	<0.01	23 (2.8)	31 (8.7)	2.12	[1.46 ; 3.08]	<0.01
**Fluoroquinolone**	48 (5)	19 (5)	0.94	[0.59 ; 1.5]	0.79	13 (10.1)	0 (0)				35 (4.2)	19 (5.4)	1.18	[0.73 ; 1.91]	0.49
**Anti MSSA**	7 (0.7)	2 (0.5)	0.60	[0.15 ; 2.39]	0.47	3 (2.3)	0 (0)				4 (0.5)	2 (0.6)	0.70	[0.18 ; 2.83]	0.62
**Anti MRSA**	20 (2.1)	14 (3.7)	1.54	[0.9 ; 2.64]	0.11	6 (4.7)	3 (10.7)	2.93	[0.87 ; 9.82]	0.08	14 (1.7)	11 (3.1)	1.41	[0.77 ; 2.59]	0.26
**MDR colonisation**	43 (4.5)	26 (6.8)	1.21	[0.81 ; 1.82]	0.35	6 (4.7)	2 (7.1)	1.22	[0.29 ; 5.16]	0.79	37 (4.4)	24 (6.8)	1.29	[0.84 ; 1.96]	0.24
**Bacteraemia**	29 (3)	19 (5)	1.47	[0.92 ; 2.34]	0.10	4 (3.1)	2 (7.1)	4.54	[1.04 ; 19.77]	0.04	25 (3)	17 (4.8)	1.35	[0.83 ; 2.2]	0.23
**Co-infection at admission**	97 (10.1)	40 (10.4)	0.89	[0.64 ; 1.24]	0.49	34 (26.4)	5 (17.9)	0.68	[0.26 ; 1.79]	0.44	63 (7.5)	35 (9.8)	1.14	[0.8 ; 1.62]	0.46

HR represenhazard Ratio for daeth at day 60.

BMI: Body Mass Index; SOFA: Sequential organ failure assessment, SAPS: simplified acute physiology score; MSSA: methicillin-susceptible *Staphylococcus aureus*, MRSA: methicillin-resistant *Staphylococcus aureus;* MDR: multi drug resistant

Table S3: Impact of Co-infection on the risk of mortality at D60, multivariate Cox model – global and per-viruses analyses according to patients' subgroups.

			**All***			**Influenza****			**SARS-CoV2*****	
	Variables	HR	HR 95% CI	*p*	HR	HR 95% CI	*p*	HR	HR 95% CI	*p*
**All**	Co-infection at admission	0.83	[0.59 ; 1.15]	0.26	0.67	[0.25 ; 1.81]	0.43	0.97	[0.68 ; 1.39]	0.88
**All**	GPC co-infection at admission	0.69	[0.41 ; 1.17]	0.20	1.37	[0.4 ; 4.72]	0.35	0.71	[0.4 ; 1.27]	0.29
	GNB co-infection at admission	1.10	[0.72 ; 1.70]	0.67	1.26	[0.29; 5.52]	0.74	1.17	[0.74; 1.84]	0.52
	No co-infection at admission	1		0.40	1		0.86	1		0.45
**Immunodepressed patients**	Co-infection at admission	0.90	[0.41 ; 2.01]	0.81	0.79	[0.18 ; 3.47]	0.75	1.03	[0.38 ; 2.79]	0.96
**Corticoids at admission**	Co-infection at admission	1.08	[0.72 ; 1.63]	0.70	2.16	[0.22 ; 21.43]	0.51	1.11	[0.73 ; 1.68]	0.64

HR represenhazard Ratio for daeth at day 60.

*Adjustment for age, chronic cardiovascular diseases, immunodepression, $PaO_2/FiO_2 < 150$, vasopressors, extrarenal purification and beta-lactamase inhibitors.

** Adjustment for age and chronic cardiovascular diseases.

*** Adjustment for age, chronic cardiovascular diseases, invasive mechanical ventilation, extrarenal purification and beta-lactamase inhibitors.

GNB: gram negative bacteria; GPC: gram positive cocci.

Table S4: Risk factors for VAP, univariate analysis, Fine-Gray subdistribution hazard model.

	**All**					**Influenza**					**SARS-CoV2**				
	No VAP	VAP	HR	HRCI	*p*	No VAP	VAP	HR	HRCI	*p*	No VAP	VAP	HR	HRCI	*p*
**Number of patients**	374	232				61	23				313	209			
**SARS-CoV2 pneumonia**	313 (83.7)	209 (90.1)	1.13	[0.71; 1.82]	0.61										
**Time from hospital admission to ICU > 2 days**	130 (34.8)	83 (35.8)	1.01	[0.78 ; 1.32]	0.92	14 (23)	6 (26.1)	1.34	[0.54 ; 3.33]	0.52	116 (37.1)	77 (36.8)	0.99	[0.75 ; 1.3]	0.92
**Age > 60 years**	235 (62.8)	137 (59.1)	0.91	[0.7 ; 1.17]	0.45	28 (45.9)	10 (43.5)	1.02	[0.46 ; 2.24]	0.96	207 (66.1)	127 (60.8)	0.89	[0.68 ; 1.17]	0.41
**Sex (male)**	257 (68.7)	173 (74.6)	1.16	[0.86 ; 1.56]	0.33	35 (57.4)	12 (52.2)	0.87	[0.39 ; 1.93]	0.73	222 (70.9)	161 (77)	1.21	[0.88 ; 1.67]	0.24
**BMI > 30 kg/m^2**	130 (34.8)	100 (43.1)	1.28	[0.98 ; 1.66]	0.07	17 (27.9)	6 (26.1)	0.77	[0.28 ; 2.11]	0.61	113 (36.1)	94 (45)	1.33	[1.01 ; 1.74]	0.04
**Chronic cardiovascular disease**	103 (27.5)	49 (21.1)	0.74	[0.53 ; 1.03]	0.08	6 (9.8)	2 (8.7)	1.19	[0.26 ; 5.45]	0.83	97 (31)	47 (22.5)	0.72	[0.51 ; 1.01]	0.06
**Chronic lung disease**	53 (14.2)	28 (12.1)	0.89	[0.6 ; 1.32]	0.56	19 (31.1)	8 (34.8)	1.57	[0.65 ; 3.79]	0.32	34 (10.9)	20 (9.6)	0.84	[0.53 ; 1.33]	0.46
**Chronic kidney disease**	40 (10.7)	14 (6)	0.59	[0.35 ; 1.01]	0.05	5 (8.2)	0 (0)				35 (11.2)	14 (6.7)	0.62	[0.36 ; 1.06]	0.08
**Immunosuppression**	68 (18.2)	16 (6.9)	0.45	[0.27 ; 0.76]	<0.01	23 (37.7)	4 (17.4)	0.48	[0.16 ; 1.45]	0.19	45 (14.4)	12 (5.7)	0.45	[0.25 ; 0.83]	0.01
**Chronic liver disease**	11 (2.9)	5 (2.2)	0.84	[0.32 ; 2.15]	0.71	2 (3.3)	3 (13)	2.83	[0.9 ; 8.91]	0.07	9 (2.9)	2 (1)	0.39	[0.09 ; 1.67]	0.21
**Diabetes**	62 (16.6)	42 (18.1)	1.12	[0.8 ; 1.58]	0.51	9 (14.8)	5 (21.7)	1.93	[0.7 ; 5.34]	0.20	53 (16.9)	37 (17.7)	1.08	[0.75 ; 1.56]	0.68
**SAPS > 40**	187 (50)	100 (43.1)	0.84	[0.65 ; 1.08]	0.18	43 (70.5)	12 (52.2)	0.65	[0.3 ; 1.44]	0.29	144 (46)	88 (42.1)	0.88	[0.67 ; 1.15]	0.34
**SOFA > 8**	96 (25.7)	54 (23.3)	0.93	[0.7 ; 1.25]	0.65	19 (31.1)	8 (34.8)	1.19	[0.51 ; 2.78]	0.69	77 (24.6)	46 (22)	0.90	[0.66 ; 1.23]	0.51
**PaO_2/FiO_2 < 150**	255 (68.2)	182 (78.4)	1.46	[1.07 ; 1.98]	0.02	34 (55.7)	15 (65.2)	1.04	[0.43 ; 2.5]	0.93	221 (70.6)	167 (79.9)	1.47	[1.05 ; 2.04]	0.02
**Invasive mechanical ventilation**	252 (67.4)	152 (65.5)	0.92	[0.7 ; 1.21]	0.55	56 (91.8)	19 (82.6)	1.03	[0.28 ; 3.8]	0.97	196 (62.6)	133 (63.6)	0.98	[0.73 ; 1.3]	0.88
**Vasopressors**	145 (38.8)	81 (34.9)	0.87	[0.65 ; 1.17]	0.35	12 (19.7)	5 (21.7)	1.01	[0.39 ; 2.59]	0.99	133 (42.5)	76 (36.4)	0.84	[0.62 ; 1.15]	0.28
Renal replacement therapy	34 (9.1)	4 (1.7)	0.21	[0.08 ; 0.55]	<0.01	7 (11.5)	0 (0)				27 (8.6)	4 (1.9)	0.24	[0.09 ; 0.63]	<0.01
**Corticoids**	168 (44.9)	128 (55.2)	1.46	[1.11 ; 1.92]	0.01	19 (31.1)	7 (30.4)	0.90	[0.4 ; 2.03]	0.81	149 (47.6)	121 (57.9)	1.50	[1.12 ; 2.01]	0.01
**Antibiotics at admission**	274 (73.3)	161 (69.4)	0.64	[0.48 ; 0.86]	<0.01	32 (52.5)	17 (73.9)	1.85	[0.68 ; 5.07]	0.23	242 (77.3)	144 (68.9)	0.56	[0.41 ; 0.76]	<0.01
**Beta-lactamase inhibitor**	63 (16.8)	37 (15.9)	0.88	[0.63 ; 1.24]	0.48	18 (29.5)	11 (47.8)	1.65	[0.74 ; 3.68]	0.22	45 (14.4)	26 (12.4)	0.77	[0.52 ; 1.14]	0.19
**Cephalosporin**	204 (54.5)	128 (55.2)	0.89	[0.69 ; 1.16]	0.40	22 (36.1)	9 (39.1)	1.08	[0.44 ; 2.67]	0.87	182 (58.1)	119 (56.9)	0.87	[0.66 ; 1.14]	0.31
**Macrolides**	127 (34)	85 (36.8)	0.99	[0.76 ; 1.29]	0.94	21 (34.4)	7 (30.4)	0.74	[0.29 ; 1.87]	0.52	106 (33.9)	78 (37.5)	1.03	[0.78 ; 1.35]	0.85
**Aminosides**	41 (11)	13 (5.6)	0.53	[0.31 ; 0.92]	0.02	8 (13.1)	2 (8.7)	0.70	[0.19 ; 2.55]	0.59	33 (10.5)	11 (5.3)	0.50	[0.28 ; 0.9]	0.02
**Fluoroquinolone**	27 (7.2)	11 (4.8)	0.61	[0.34 ; 1.1]	0.10	4 (6.6)	2 (8.7)	1.33	[0.31 ; 5.78]	0.70	23 (7.3)	9 (4.3)	0.59	[0.31 ; 1.12]	0.11
**Anti MSSA**	8 (2.1)	0 (0)				3 (4.9)	0 (0)				5 (1.6)	0 (0)			
**Anti MRSA**	17 (4.5)	4 (1.7)	0.47	[0.18 ; 1.25]	0.13	6 (9.8)	1 (4.3)	0.56	[0.07 ; 4.4]	0.58	11 (3.5)	3 (1.4)	0.49	[0.16 ; 1.46]	0.20
**MDR colonisation**	19 (5.1)	20 (8.7)	1.48	[0.92 ; 2.37]	0.11	3 (4.9)	4 (17.4)	3.16	[1.25 ; 7.98]	0.01	16 (5.1)	16 (7.7)	1.35	[0.79 ; 2.3]	0.27
**Bacteraemia**	16 (4.3)	9 (3.9)	0.98	[0.49 ; 1.94]	0.95	5 (8.2)	0 (0)				11 (3.5)	9 (4.3)	1.25	[0.63 ; 2.49]	0.53
**Co-infection at admission**	56 (15)	31 (13.4)	0.83	[0.57 ; 1.2]	0.32	18 (29.5)	9 (39.1)	0.96	[0.36 ; 2.56]	0.94	38 (12.1)	22 (10.5)	0.76	[0.49 ; 1.18]	0.22

VAP: Ventilator Associated Pneumonia; BMI: Body Mass Index; SOFA: Sequential organ failure assessment, SAPS: simplified acute physiology score; MSSA: methicillin-susceptible *Staphylococcus aureus*, MRSA: methicillin-resistant *Staphylococcus aureus;* MDR: multi drug resistant

Table S5: Impact of co-infection on the risk of subsequent VAP among the patients at risk of VAP – multivariate survival analysis

Cohort	Comparisons	SubHR	SubHR 95% CI	p
**All cohort***	RespCoBact vs no RespCoBact	0.82	[0.57; 1.18]	0.30
	SARS-CoV2 vs Influenza	1.08	[0.66; 1.75]	0.76
**All Cohort***	Influenza: RespCoBact vs no RespCoBact	1.11	[0.46; 2.66]	0.82
	SARS-CoV2: RespCoBact vs no RespCoBact	0.86	[0.52; 1.4]	0.54
	No RespCoBact: SARS-CoV2 vs Influenza	1.19	[0.67; 2.13]	0.55
	RespCoBact: SARS-CoV2 vs Influenza	0.93	[0.41; 2.07]	0.85
**Influenza****	RespCoBact vs no RespCoBact	0.78	[0.28 ; 2.14]	0.62
**SARS-CoV2***	RespCoBact vs no RespCoBact	0.75	[0.49 ; 1.13]	0.17

*adjustment for chronic kidney disease, acute respiratory failure on admission, antimicrobial therapy on admission, steroids on admission
**adjustment for MDR colonisation.

VAP: Ventilator Associated Pneumonia; RespCoBact: Respiratory Bcterial Co-Infection on admission

Table S6: Risk factors for co-infection, univariate analysis, logistic regression.

	Influenza					SARS-CoV2				
	No ResCoBact N=118	ResCoBact N=39	OR	ORCI	p	No ResCoBact N=1094	ResCoBact N=98	OR	ORCI	p
**Time from hospital admission to ICU > 2 days**	32 (27.1)	6 (15.4)	0.49	[0.19 ; 1.28]	0.14	447 (40.9)	41 (41.8)	1.04	[0.68 ; 1.58]	0.85
**Age > 60 years**	58 (49.2)	21 (53.8)	1.21	[0.58 ; 2.49]	0.61	683 (62.4)	61 (62.2)	0.99	[0.65 ; 1.52]	0.97
**Sex (male)**	71 (60.2)	22 (56.4)	0.86	[0.41 ; 1.78]	0.68	790 (72.2)	74 (75.5)	1.19	[0.73 ; 1.92]	0.48
**BMI > 30 kg/m^2**	40 (33.9)	5 (12.8)	0.29	[0.1 ; 0.79]	0.02	425 (38.8)	38 (38.8)	1.00	[0.65 ; 1.52]	0.99
**Chronic cardiovascular disease**	20 (16.9)	2 (5.1)	0.26	[0.06 ; 1.19]	0.08	275 (25.1)	31 (31.6)	1.38	[0.88 ; 2.16]	0.16
**Chronic lung disease**	37 (31.4)	12 (30.8)	0.97	[0.44 ; 2.13]	0.95	123 (11.2)	10 (10.2)	0.90	[0.45 ; 1.77]	0.75
**Chronic kidney disease**	16 (13.6)	2 (5.1)	0.34	[0.08 ; 1.57]	0.17	98 (9)	10 (10.2)	1.15	[0.58 ; 2.29]	0.68
**Immunodepression**	51 (43.2)	8 (20.5)	0.34	[0.14 ; 0.8]	0.01	132 (12.1)	14 (14.3)	1.21	[0.67 ; 2.2]	0.52
**Chronic liver disease**	6 (5.1)	1 (2.6)	0.49	[0.06 ; 4.21]	0.52	20 (1.8)	6 (6.1)	3.50	[1.37 ; 8.94]	<0.01
**Diabetes**	21 (17.8)	5 (12.8)	0.68	[0.24 ; 1.94]	0.47	165 (15.1)	19 (19.4)	1.35	[0.8 ; 2.3]	0.26

BMI: Body Mass Index; ResCoBact: Respiratory bacterial co infection on admission
